# Global trends in thermal burn burden, 1990–2021: a comprehensive analysis for the global burden of disease study 2021

**DOI:** 10.3389/fpubh.2025.1631138

**Published:** 2025-07-30

**Authors:** Yuke Zhang, Qingsong Chen, Ru Wang, Linfen Guo, Peiyu Li, Ke Deng, Haitao Xiao, Xuewen Xu

**Affiliations:** ^1^Department of Plastic and Burn Surgery, West China Hospital, Sichuan University, Chengdu, China; ^2^School of Microelectronics and Communication Engineering of Chongqing University, Chongqing University Central Hospital (Chongqing Emergency Medical Center), Chongqing, China

**Keywords:** thermal burns, global burden of disease, incidence, prevalence, years lived with disability

## Abstract

**Background:**

The purpose of this study is to explore the global burden of thermal burns from 1990 to 2021 and to predict the trends in thermal burn burden up to 2040.

**Methods:**

We utilized data from the Global Burden of Disease Study 2021 to assess the incidence, prevalence, and years lived with disability (YLDs) of thermal burns at the global, regional, and national levels. Decomposition analysis was performed to quantify the relative contributions of epidemiological changes, age structure shifts, and population growth to the thermal burn burden. Frontier analysis was employed to evaluate the potential improvements in the thermal burn burden that could be achieved given a country’s development status. A Bayesian age-period-cohort model was used to predict burden trends up to 2040.

**Results:**

In 2021, the global numbers of thermal burn incident cases and YLDs were approximately 6.19 million and 2.67 million, respectively, representing decreases of 9.44 and 11.86% from 1990. The prevalent cases were around 104.76 million, an increase of 11.38% from 1990. The age-standardized rates of incidence, prevalence, and YLDs all showed declines. Decomposition analysis indicated that population growth is the primary factor hindering the reduction of the thermal burn burden. Frontier analysis suggested that countries at various development levels have the potential to improve the thermal burn burden. By 2040, the number of thermal burn incident cases is predicted to decline, while the numbers of prevalent cases and YLDs will increase.

**Conclusion:**

The global burden of thermal burns has been partially alleviated, but it will continue to persist. For low socio-demographic index countries, it is necessary to adopt targeted healthcare measures tailored to their specific circumstances alongside social development to achieve optimal management of thermal burns.

## 1 Introduction

Burns are a common and serious public health problem worldwide. According to the World Health Organization, approximately 180,000 people die from burns each year globally (1). From an etiological perspective, burns can be classified into various types, such as thermal burns, chemical burns, electrical burns, and radiation burns (2). Among these, thermal burns are the most common type, primarily caused by contact with flames, steam, and hot liquids. Severe burns not only cause fatal physiological damage to patients, including tissue necrosis, infection, shock, and disability, but can also lead to long-term psychological and social issues, such as depression, anxiety, alcoholism, and post-traumatic stress disorder (3, 4).

Recent studies indicate that the global incidence of burns has generally shown a declining trend (5). However, significant regional disparities in burn burden persist, which are closely related to variations in economic development, healthcare infrastructure, and preventive measures across regions. For instance, studies focusing on low- and middle-income countries have found that burn incidence in these regions is substantially higher than in high-income countries, while inadequate medical resources contribute to elevated mortality rates among burn patients (6, 7). Additionally, burn treatment is complex and costly. In high-income countries, the average medical cost per burn patient is $88,218, and burns often result in indirect economic losses due to limited mobility and reduced productivity (8, 9). Given the substantial public health and socioeconomic burden imposed by burns, a comprehensive understanding of the burden and evolving trends across regions with different sociodemographic profiles is critical.

The Global Burden of Disease (GBD) database is widely used to assess the global health impact of diseases and injuries. It provides a robust framework for analyzing the epidemiology and health burden of thermal burns, thereby informing evidence-based policy decisions (10). In this study, we utilized data from the GBD 2021 Study to analyze trends in thermal burn incidence, prevalence, years lived with disability (YLDs), and corresponding age-standardized rates (ASRs) across countries and regions from 1990 to 2021. Furthermore, decomposition analysis and frontier analysis were conducted to identify key drivers of thermal burn burden and evaluate potential mitigation strategies. Finally, we projected trends in thermal burn burden up to 2040 using existing data. Our findings aim to elucidate the heterogeneous burden of thermal burns globally and provide actionable insights for healthcare policymakers.

## 2 Methods

### 2.1 Data source

The data used in this study were sourced from the GBD Study 2021, a comprehensive epidemiological database that aggregates information from 100,983 data sources, including vital registration systems, censuses, household surveys, disease registries, and healthcare facility records, to estimate the global, regional, and national burden of 371 diseases and injuries (10). We extracted incidence, prevalence, and YLDs data for thermal burns across countries and regions from 1990 to 2021. These data were utilized for descriptive analysis after consolidation.

### 2.2 Trends analysis

To comprehensively understand the changing burden of thermal burns, we explored trends in the number of thermal burn cases and ASRs. For the numbers of incident cases, prevalent cases, and YLDs, we calculated their percentage changes based on case counts in 1990 and 2021. For ASRs of incidence (ASIR), prevalence (ASPR), and YLDs (ASYR), the estimated average percent change (EAPC) was used to measure trends from 1990 to 2021. To calculate EAPC, we first fitted the data using a regression model with the following equation: Y=α+βX+ε . where Y represents the natural logarithm of ASRs, αis the intercept, βdenotes the annual change rate of the natural logarithm of ASRs, X corresponds to the calendar year, and *ε* represents the error term. The estimated average percent change was then computed using the formula: $\text{EAPC}=100\times (e^{\beta }-1)$ . In the EAPC formula, e denotes the base of the natural logarithm, used to convert the regression coefficient from a logarithmic scale back to a percentage change (11).

### 2.3 The relationship between socio-demographic index (SDI) and the burden of thermal burns

SDI is a composite indicator of development status correlated with health outcomes. It is calculated based on fertility rate, income per capita, and level of education, ranging from 0 to 1. An SDI of 0 indicates the lowest level of development related to health, whereas an SDI of 1 represents the highest level of health-related development (12). To understand the relationship between SDI and the burden of thermal burns, we used Pearson correlation analysis to evaluate the correlation between SDI and the ASRs of incidence, prevalence, and YLDs of thermal burns across 204 countries and territories, and 21 GBD regions.

### 2.4 Decomposition analysis

To gain a deeper understanding of the factors contributing to changes in the incident cases, prevalent cases, and YLDs of thermal burns from 1990 to 2021, the decomposition analysis was performed. This analysis separated the influences of population, age structure, and incidence rate (defined here as the epidemiological changes) on the incidence burden (13, 14). The decomposition formula is as follows:

$${\text{Incidence}}_{\mathrm{ay},\mathrm{py},\mathrm{ey}}=\sum_{i=1}^{n}(a_{i,y}*p_{y}*e_{i,y})$$

where Incidence_ay, py, ey_ represents the number of incident cases calculated based on population, age structure, and Incidence rate; *a_i,y_* denotes the proportion of the population in the *i*-th age group in the specified year *y*; *p_y_* indicates the total population in the specified year *y*; *e_i,y_* represents the incidence rate for the *i*-th age group in the specified year *y*; and *n* denotes the number of age groups. Using this formula, we can substitute incidence rate with prevalence or YLDs rates to perform decomposition analysis on the numbers of prevalent cases or YLDs.

### 2.5 Frontier analysis

We employed frontier analysis to benchmark the burden of thermal burns, comparing each country against the best-performing counterparts (15). The frontier line of ASRs of incidence, prevalence, and YLDs represents the lowest burden of thermal burns that each country could achieve under its SDI conditions. The gap between a country’s actual burden and the frontier line is referred to as the effective difference. A larger effective difference indicates that a country has unrealized opportunities to reduce the burden of thermal burns given its current level of development. In our study, we used 1,000 bootstrapped samples of the data and calculated the mean ASRs of incidence, prevalence, and YLDs for each SDI value. A locally weighted regression with a local polynomial degree of 1 and a span of 0.2 was then developed to generate a smoothed frontier. Countries with super-efficient performance were excluded to prevent outliers from affecting the analysis.

### 2.6 Projection of thermal burn burden

To calculate and predict the future burden of thermal burns, we utilized a Bayesian Age-Period-Cohort (BAPC) analysis, which employs integrated nested Laplace approximations for more accurate trend projections (16). In our analysis, we sourced population estimates from the Institute for Health Metrics and Evaluation, which provides data categorized by year, age, and sex up to the year 2,100.

### 2.7 Statistical analysis

The case numbers and ASRs of incidence, prevalence, and YLDs obtained from the GBD Study 2021 were presented as absolute values along with their 95% uncertainty intervals. The trends in ASRs were expressed using the EAPC and the corresponding 95% CI. In the correlation analysis between ASRs and SDI, a *p*-value of less than 0.05 was considered statistically significant. All analyses in this study were conducted using R version 4.3.1.

## 3 Results

### 3.1 Global trends in the burden of thermal burns

**Figure 1:**
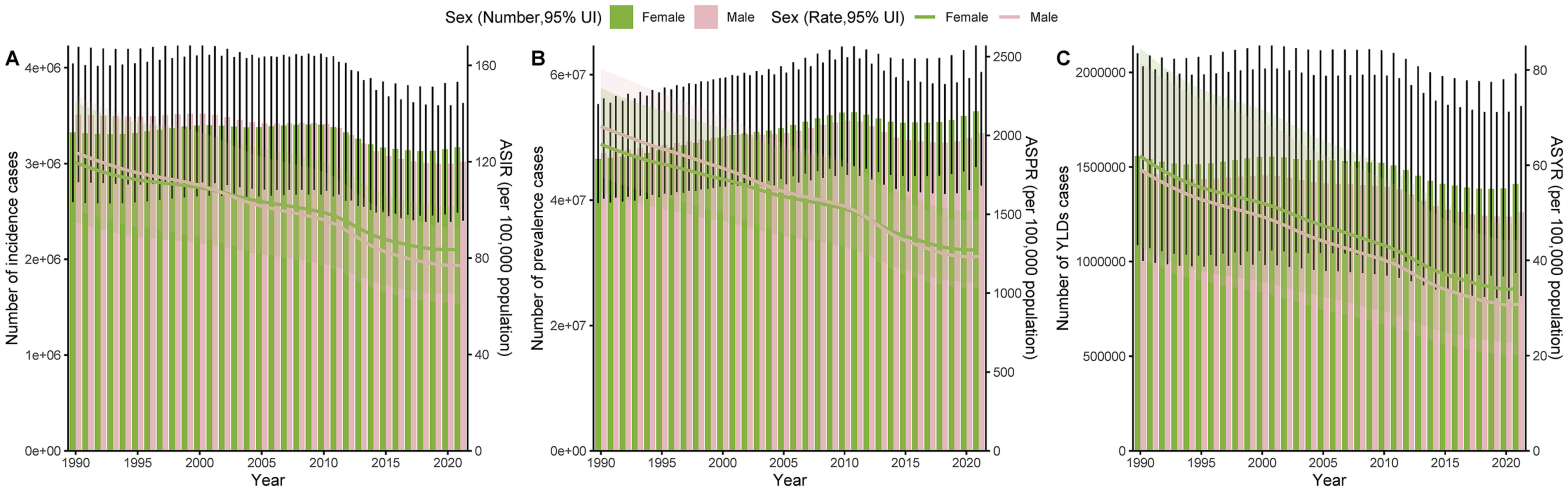
Trends in case numbers and ASRs of incidence **(A)**, prevalence **(B)**, and YLDs **(C)** of thermal burns by gender from 1990 to 2021.

In 2021, the global incident cases of thermal burns were approximately 6.19 million, representing a 9.44% decrease compared to 1990. The number of YLDs was about 2.67 million, a reduction of 11.86% from 1990. However, the number of prevalent cases increased from approximately 94.06 million to 104.76 million, reflecting an 11.38% rise over this period. The ASRs of incidence, prevalence, and YLDs all exhibited declining trends, with EAPCs of −1.40, −1.56, and −2.06, respectively. Despite the overall downward trends in ASRs, the decline in the thermal burn burden among females was slower than that among males (Figures 1A–C, Supplementary Tables S1, S2).

### 3.2 The burden of thermal burns by specific age groups

**Figure 2:**
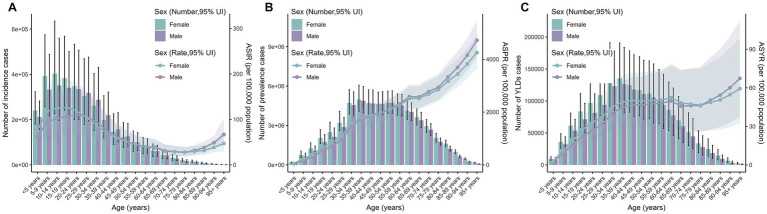
Age-specific case numbers and ASRs of incidence **(A)**, prevalence **(B)**, and YLDs **(C)** for thermal burns in 2021.

In 2021, the highest number of incident cases of thermal burns globally occurred in the 10–14 age group, while the highest numbers of prevalent cases and YLDs were observed in the 35–39 age group. Compared to 1990, the number of incident cases, prevalent cases, and YLDs had significantly decreased in children but increased in older adults. In terms of ASRs, the highest ASIR in 2021 was observed in adolescents aged 15–19, whereas the highest ASPR and ASYR were found in individuals aged 95 years and older. Compared to 1990, ASRs of incidence, prevalence, and YLDs had declined across all age groups, with the most pronounced decreases in children under 5 years and the smallest reductions in those aged 95 years and older. Furthermore, the distribution of ASIR in both 1990 and 2021 exhibited a bimodal pattern, peaking in children and adolescents, declining with age, and rising again after 70 years (Figures 2A–C, Supplementary Tables S3, S4).

### 3.3 Trends in the burden of thermal burns across different SDI regions

In 2021, the middle SDI quintile had the highest burden of thermal burns, with approximately 1.62 million incident cases and 0.74 million YLDs, while the high SDI quintile reported the highest number of prevalent cases at 30.14 million. Compared to 1990, both the high SDI and high-middle SDI quintiles showed significant reductions in incident cases, prevalent cases, and YLDs by 2021. In contrast, the low SDI and low-middle SDI quintiles had the lowest case numbers overall, but these were increasing rapidly (Supplementary Table S1). For ASRs, the high SDI quintile exhibited the highest ASIR (141.85 per 100,000 population) and ASPR (2097.44 per 100,000 population). However, this quintile also demonstrated the steepest declines in ASIR and ASPR from 1990 to 2021, with EAPCs of −1.66 and −1.58, respectively. The low SDI regions had the highest ASYR at 47.21 per 100,000 population, though their ASYR showed a modest downward trend (EAPC = −1.26) over the same period (Supplementary Table S2).

### 3.4 National trends in the burden of thermal burns

**Figure 3:**
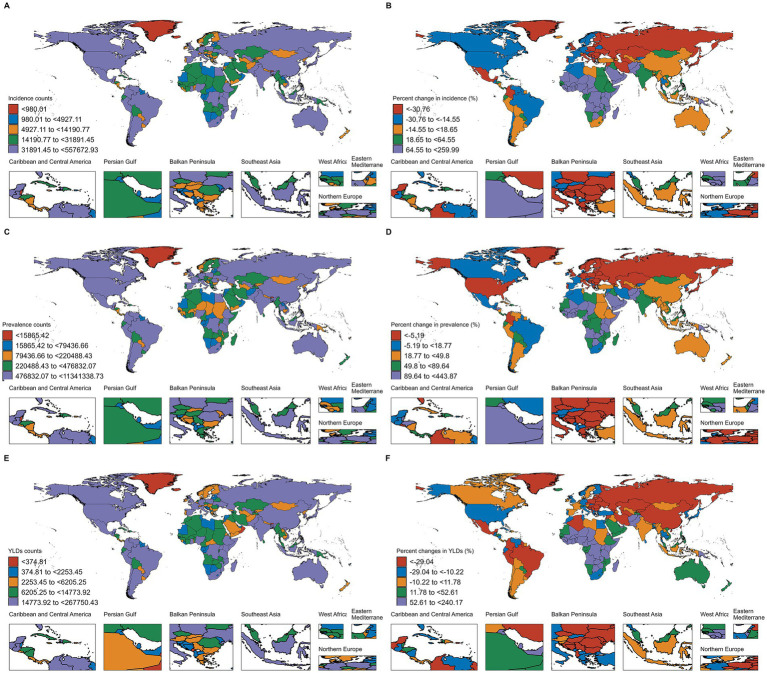
Number of incident cases of thermal burns in 2021 **(A)**, and percentage change in cases from 1990 to 2021 **(B)**. Number of prevalent cases of thermal burns in 2021 **(C)**, and percentage change in cases from 1990 to 2021 **(D)**. Number of YLDs of thermal burns in 2021 **(E)**, and percentage change in cases from 1990 to 2021 **(F)**.

**Figure 4:**
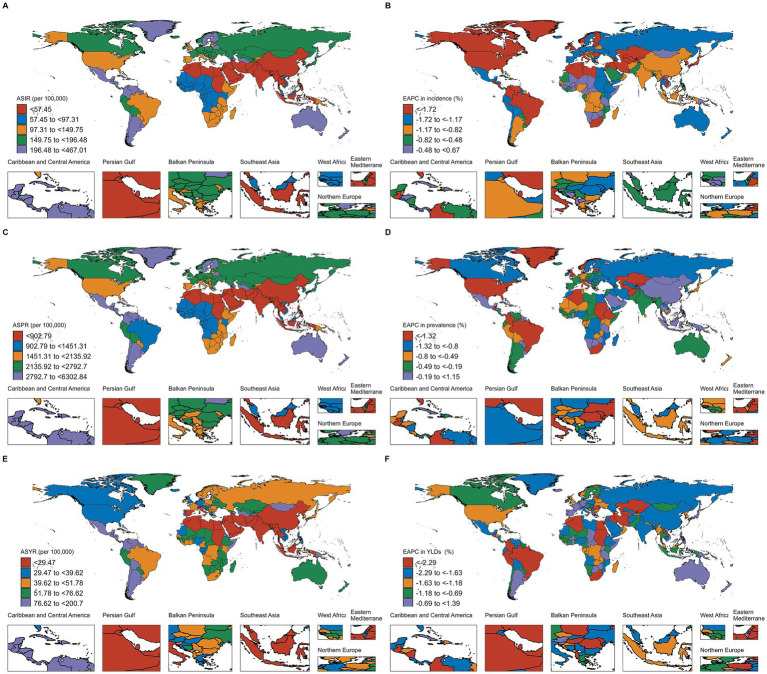
ASRs of incidence of thermal burns in 2021 **(A)**, and EAPC from 1990 to 2021 **(B)**. ASRs of prevalence of thermal burns in 2021 **(C)**, and EAPC from 1990 to 2021 **(D)**. ASRs of YLDs of thermal burns in 2021 **(E)**, and EAPC from 1990 to 2021 **(F)**.

In terms of absolute case numbers, India, China, and the United States of America had the highest burden of thermal burns globally. Compared to 1990, the United States of America showed reductions of 29.59% in incident cases, 14.06% in prevalent cases, and 16.08% in YLDs. China experienced a 12.98% decrease in incident cases and a 34.39% decline in YLDs, but a 35.49% increase in prevalent cases. Notably, India demonstrated rising trends across all three metrics, with 23.88, 53.93, and 5.76% increases in incident cases, prevalent cases, and YLDs, respectively (Figure 3, Supplementary Table S1). For ASRs, Chile had the highest ASIR (693.70 per 100,000 population) and ASPR (6302.84 per 100,000 population), while Haiti exhibited the highest ASYR (200.7 per 100,000 population). Among all countries, Tuvalu showed the most substantial declines in ASIR, ASPR, and ASYR from 1990 to 2021, with EAPCs of −11.12, −10.06, and −11.21, respectively (Figure 4, Supplementary Table S2).

### 3.5 Decomposition analysis

**Figure 5:**
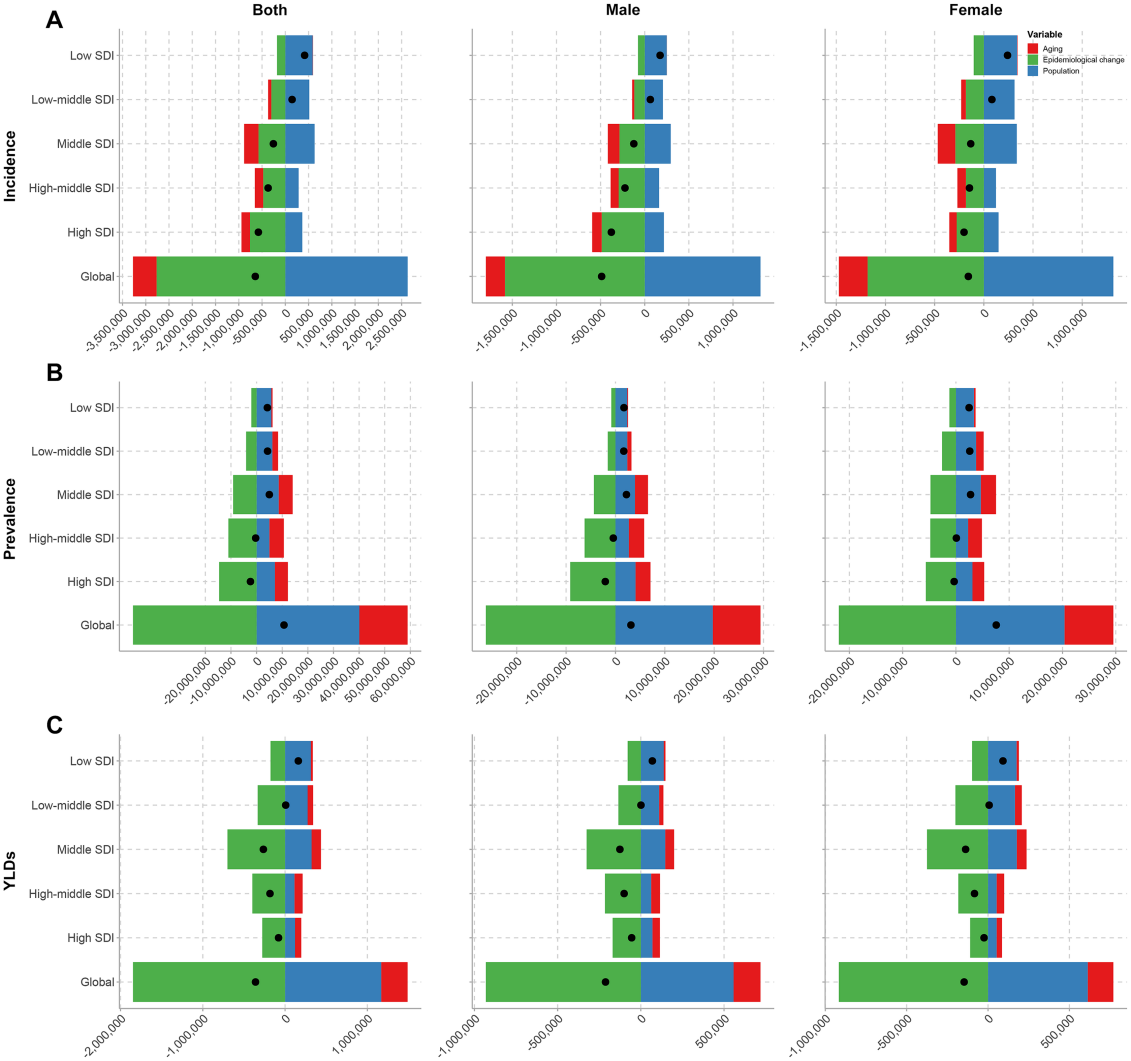
Decomposition analysis of trends in the incidence **(A)**, prevalence **(B)**, and YLDs **(C)** of thermal burns from 1990 to 2021, by sex, at the global level and across five SDI regions.

Over the 31-year period (1990–2021), epidemiological changes contributed most substantially to the reduction in thermal burn incidence, accounting for 428.73% of the total decline. Similar trends were observed across sex groups (Figure 5A, Supplementary Table S5). For thermal burn prevalence, however, while epidemiological changes (−450.43%) contributed to improvements, their effect was outweighed by the combined impacts of population growth (374.54%) and aging (175.89%) (Figure 5B, Supplementary Table S6). In contrast, epidemiological changes (514.58%) dominated the reduction in global YLDs, counteracting the negative effects of population growth (−325.56%) and aging (−89.02%) (Figure 5C, Supplementary Table S7).

### 3.6 Relationship between the burden of thermal burns and SDI

**Figure 6:**
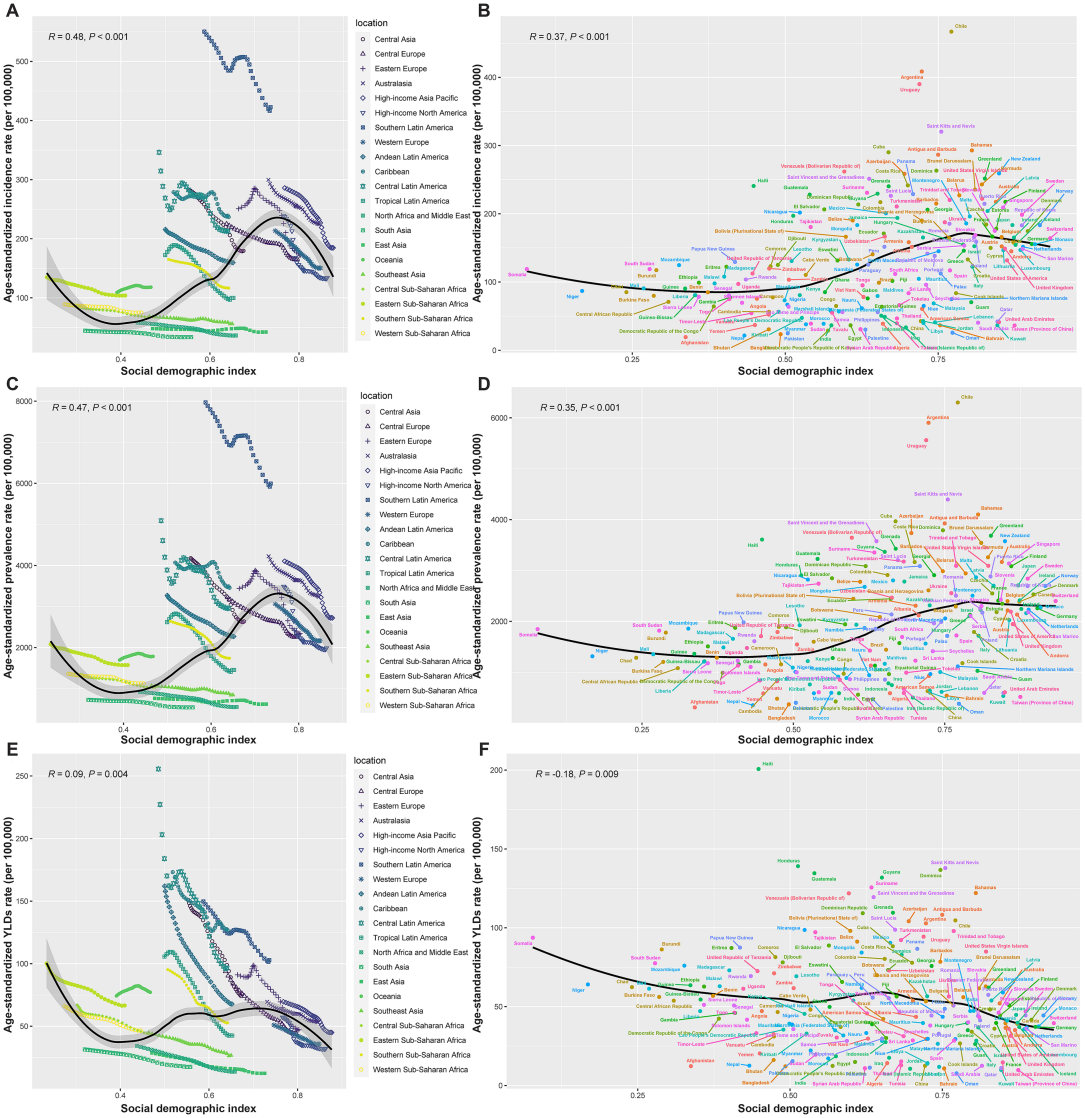
Pearson correlation between SDI and ASRs of incidence **(A,B)**, prevalence **(C,D)**, and YLDs **(E,F)** for 21 GBD regions and 204 countries.

Figure 6 illustrates the correlation between SDI and ASRs of thermal burns across regions and countries. The ASIR initially decreased with rising SDI but began to increase at an SDI of approximately 0.4, peaked around 0.75, and subsequently declined. Overall, ASIR demonstrated a positive correlation with SDI (Figures 6A,B). A similar trend was observed for ASPR (Figures 6C,D). In contrast, the correlation between SDI and ASYR was weak. Among the 21 GBD regions, SDI showed a positive correlation with ASYR (R = 0.09, *p* = 0.004). However, analysis across 204 countries and territories revealed a negative correlation between SDI and ASYR (R = −0.18, *p* = 0.009) (Figures 6E,F).

### 3.7 Frontier analysis

**Figure 7:**
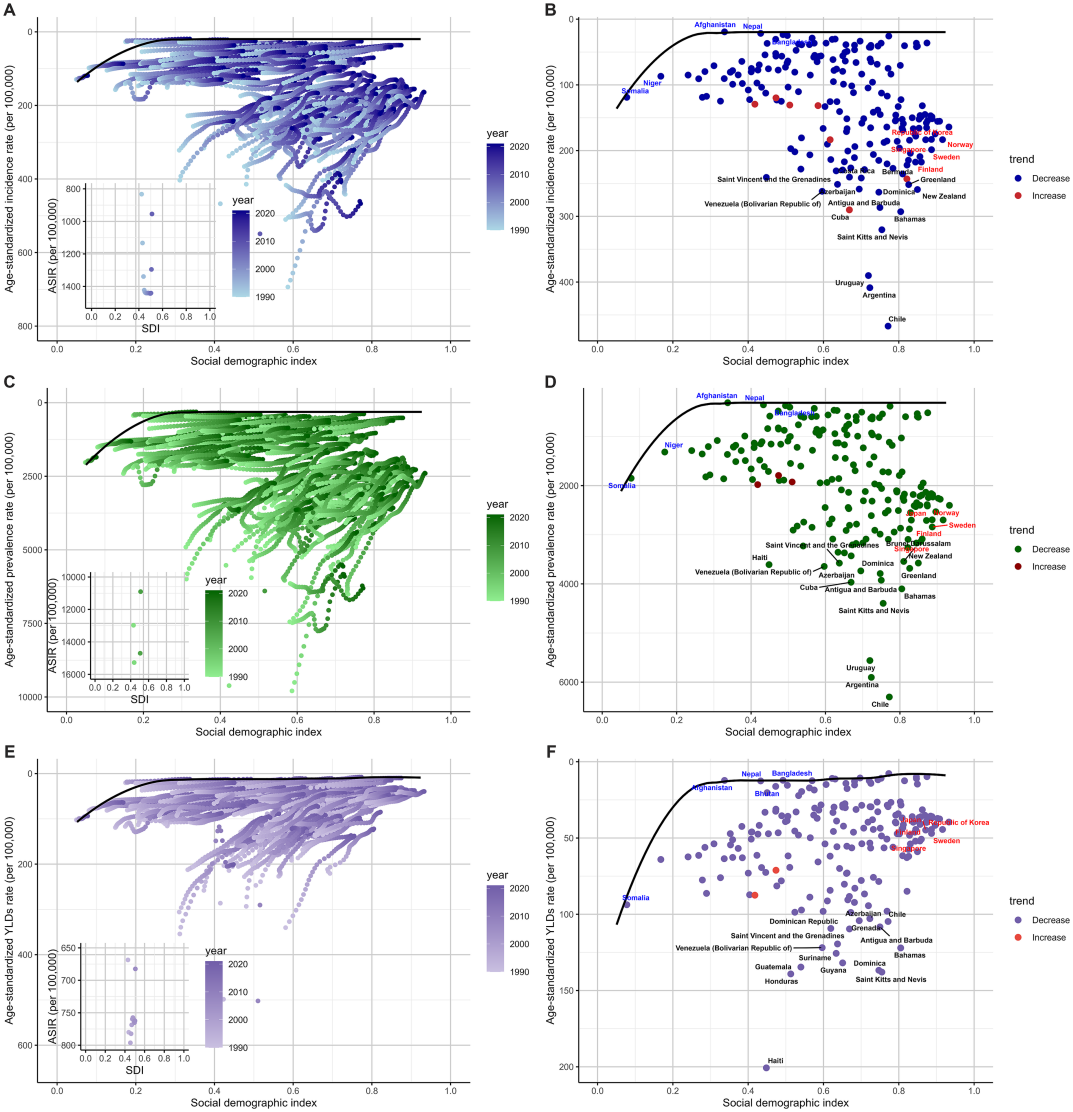
Frontier analysis based on SDI and ASRs of incidence **(A,B)**, prevalence **(C,D)**, and YLDs **(E,F)**.

The results indicated that as SDI increased, the effective differences for ASIR and ASPR generally expanded, with the most pronounced disparities observed in high-middle SDI countries. Countries such as Chile, Argentina, Uruguay, Saint Kitts and Nevis, and the Bahamas were positioned far from the frontier, demonstrating ASIR and ASPR substantially above the expected values for their SDI levels (Figures 7A–D, Supplementary Tables S8, S9). For ASYR, when SDI was below 0.8, the effective difference increased with rising SDI. However, beyond an SDI of 0.8, the effective difference for ASYR decreased significantly. The most notable effective differences for ASYR were observed in low-middle SDI, middle SDI and high-middle SDI countries, such as Haiti, Saint Kitts and Nevis, Honduras, Dominica, and Guatemala (Figures 7E,F, Supplementary Table S10).

### 3.8 Predictive analysis of the burden of thermal burns to 2040

**Figure 8:**
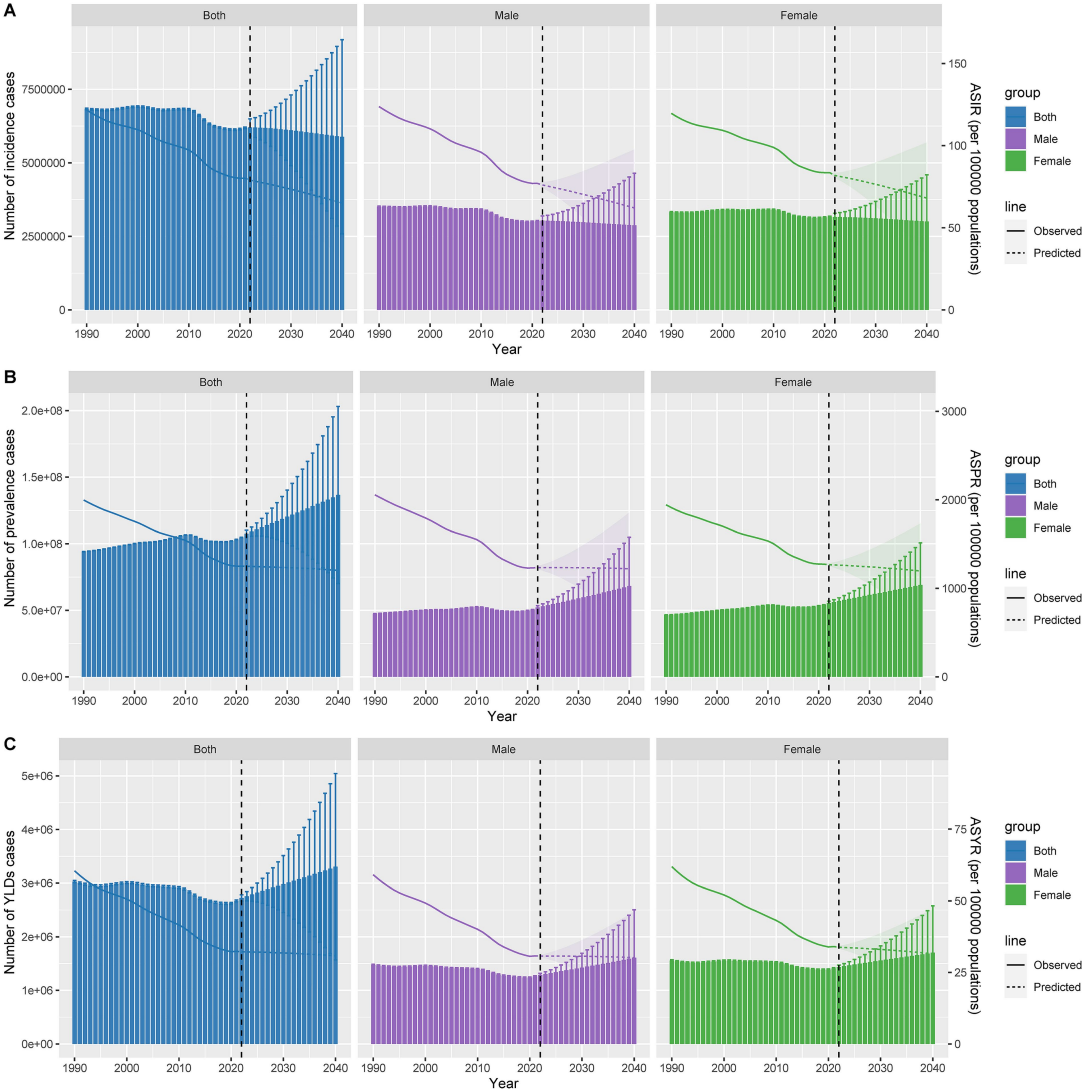
Predicted case numbers and ASRs of incidence **(A)**, prevalence **(B)**, and YLDs **(C)** for thermal burns up to 2040.

The results indicated that the number of incident cases would decline gradually by 2040, while the numbers of prevalent cases and YLDs were projected to increase steadily. The ASIR was predicted to show a relatively significant decrease, whereas the ASPR and ASYR were expected to decline minimally over the same period (Figures 8A–C).

## 4 Discussion

Our findings reveal that, similar to patterns observed three decades ago, children, adolescents, and the older adult continue to exhibit higher ASIR. Children’s heightened susceptibility to burns is driven by inherent curiosity and limited awareness of danger (17, 18), while older adult individuals face increased risks due to age-related declines in mobility, judgment, and coordination (19). Although children still account for a substantial proportion of global thermal burn cases, the number of incident cases among children, particularly those under five years old, has declined significantly since 1990. This improvement is likely attributable to enhanced community education and increased preventive awareness among caregivers (20). In contrast, although the absolute number of incident cases among the older adult remains low, the aging population has led to a marked rise in older adult burn cases. Additionally, age-related physiological decline complicates clinical management in this group, often resulting in higher mortality rates (21). Given the severe health consequences and medical burden of burns in the older adult, targeted interventions are urgently needed.

Globally, prevalent cases of thermal burns increased by 11.38% from 1990 to 2021, while incident cases and YLDs showed slight declines, as did ASRs of incidence, prevalence, and YLDs. Notably, despite substantial reductions in incident and prevalent cases in high SDI countries, these regions still ranked second and first globally in incident and prevalent cases, respectively. Conversely, low SDI countries reported the lowest case numbers across SDI quintiles but faced rapidly escalating burdens. In many low SDI nations, such as Somalia, Niger, Chad, and Mali, armed conflicts and political instability exacerbated burn risks, while inadequate healthcare infrastructure contributed to high mortality (22, 23). Although GBD 2021 lacks direct mortality data for thermal burns, our study highlights the highest ASYR in low SDI regions, reflecting poor healthcare capacity to address burn-related disability (24).

At the national level in 2021, India had the highest numbers of incident cases and YLDs of thermal burns, while China had the highest number of prevalent cases. Compared to 1990, the number of prevalent cases in China increased, whereas the numbers of incident cases and YLDs decreased. In contrast, India experienced increases in incident cases, prevalent cases, and YLDs. Decomposition analysis revealed that population growth was the primary driver of rising incidence, prevalence, and YLDs of thermal burns. As the two most populous countries globally, India and China differed in population dynamics, with India’s higher population growth rate leading it to surpass China as the world’s most populous nation in 2023. Given India’s escalating thermal burn burden over the past 31 years, sustained population growth is expected to exacerbate healthcare challenges (25). In terms of ASRs, Chile recorded the highest ASIR and ASPR of thermal burns among all countries. Recent decades saw a sharp rise in fire activity in Chile, driven by climate change and extreme weather, with approximately 6,000 recorded fires annually (26). This is likely a major contributing factor to the burden of thermal burns in Chile. Notably, despite these risks, Chile achieved reductions in both incident cases and ASIR compared to 1990, possibly due to enhanced public fire prevention awareness and effective healthcare interventions. The divergent burden patterns across regions and countries underscore the need for tailored health strategies to address context-specific challenges.

Our study indicated that SDI was positively correlated with the ASRs of incidence and prevalence. One possible explanation was that as SDI increased, the use of devices and fuels became more widespread. However, issues such as poor equipment quality, fuel instability, and improper usage likely contributed to higher burn occurrence (27). Additionally, societal progress led to the development of industries like services, construction, chemicals, and smelting, which raised the proportion of work-related burns. A previous report noted that from 2009 to 2016, 17% of severe burns in Australia and New Zealand were work-related, with the highest proportions occurring in trade, service sectors, and industrial or construction sites (28). At the national level, SDI showed a weak negative correlation with ASYR, potentially because higher SDI countries generally had better healthcare systems and medical access. In contrast, at the regional level, SDI exhibited a weak positive correlation with ASYR, suggesting that genetic or environmental factors might also influence this relationship. These findings underscored the need to enhance public awareness and occupational burn prevention measures while improving medical standards alongside societal development.

The frontier analysis, based on SDI and ASRs of thermal burns, depicted trends in the disease burden across countries from 1990 to 2021. The results revealed that although the ASRs of incidence, prevalence, and YLDs of thermal burns showed a declining trend in most countries, many countries at different development levels still had significant distances from the frontier, indicating unrealized opportunities to close the gap of ASRs. Notably, some low SDI countries are close to the aspirational benchmark of the frontier. These countries can serve as models for reducing the burden of thermal burns under limited social and healthcare resources. In contrast, some high SDI countries, such as Finland and New Zealand, performed poorly. This discrepancy highlights the multifaceted nature of healthcare outcomes, indicating that while higher SDI brings better healthcare resources, other potential factors such as environment, education level, and living habits may also play important roles in regulating disease burden (29).

Several limitations in our study need to be noted. First, the GBD data comes from various countries, and the accuracy and consistency of this data may vary. Although GBD collaborators used advanced statistical modeling methods to standardize data and mitigate biases, the reliance on model data means there is a potential for distortion in the results. Additionally, the GBD 2021 categorizes burns as natural injuries, and while burns can be fatal, the data available to us only includes incidence, prevalence, and YLDs. Therefore, our study only evaluates the burden of non-fatal burns.

## 5 Conclusion

Our study provides a comprehensive interpretation of the burden of thermal burns at global, regional, and national levels, and predicts future trends in disease burden. This enhances our understanding of the epidemiology of thermal burns and emphasizes the necessity of targeted medical interventions. One issue faced by low SDI regions is a significant increase in the number of burn patients, which will inevitably increase the local healthcare burden. Improving healthcare accessibility to ensure patients receive the most basic treatments is crucial. High SDI regions generally possess sufficient and high-quality medical facilities, where reducing disabilities caused by burns is the highest priority.

## Data Availability

The original contributions presented in the study are included in the article/Supplementary material, further inquiries can be directed to the corresponding authors.
